# Beliefs related to adherence to oral antidiabetic treatment according to the Theory of Planned Behavior^1^

**DOI:** 10.1590/0104-1169.3578.2448

**Published:** 2014

**Authors:** Fernanda Freire Jannuzzi, Roberta Cunha Matheus Rodrigues, Marilia Estevam Cornélio, Thaís Moreira São-João, Maria Cecília Bueno Jayme Gallani

**Affiliations:** 2 Doctoral student, Faculdade de Enfermagem, Universidade Estadual de Campinas, Campinas, SP, Brazil; 3 PhD, Associate Professor, Faculdade de Enfermagem, Universidade Estadual de Campinas, Campinas, SP, Brazil; 4 PhD, Assistant Professor, Departamento de Medicina e Enfermagem, Universidade Federal de Viçosa, Viçosa, MG, Brazil; 5 Post-doctoral fellow, Faculdade de Enfermagem, Universidade Estadual de Campinas, Campinas, SP, Brazil; 6 PhD, Associate Professor, Faculdade de Enfermagem, Universidade Estadual de Campinas, Campinas, SP, Brazil. Full Professor, Faculté des sciences infirmières, Université Laval, Québec, Canada

**Keywords:** Medication Adherence, Behavior, Diabetes Mellitus, Nursing

## Abstract

**OBJECTIVE:**

to identify salient behavioral, normative, control and self-efficacy beliefs related to the behavior of adherence to oral antidiabetic agents, using the Theory of Planned Behavior.

**METHOD:**

cross-sectional, exploratory study with 17 diabetic patients in chronic use of oral antidiabetic medication and in outpatient follow-up. Individual interviews were recorded, transcribed and content-analyzed using pre-established categories.

**RESULTS:**

behavioral beliefs concerning advantages and disadvantages of adhering to medication emerged, such as the possibility of avoiding complications from diabetes, preventing or delaying the use of insulin, and a perception of side effects. The children of patients and physicians are seen as important social references who influence medication adherence. The factors that facilitate adherence include access to free-of-cost medication and taking medications associated with temporal markers. On the other hand, a complex therapeutic regimen was considered a factor that hinders adherence. Understanding how to use medication and forgetfulness impact the perception of patients regarding their ability to adhere to oral antidiabetic agents.

**CONCLUSION:**

medication adherence is a complex behavior permeated by behavioral, normative, control and self-efficacy beliefs that should be taken into account when assessing determinants of behavior.

## Introduction

The use of non-pharmacological and pharmacological treatment is recommended to control and facilitate the management of Diabetes Mellitus (DM). Hence, special attention has been paid to factors related to medication adherence, since patients with chronic diseases, the emergence of which are asymptomatic, are more likely not to adhere to medication^(^ 1 ^)^. It is known that despite considerable technological advancements achieved in regard to the diagnosis and treatment of DM patients, a large number of people do not adhere to their recommended treatment^(^ 2 ^-^ 3 ^)^.

In general, medication adherence is seen as a measure in which patients follow instructions for the treatments prescribed^(^ 4 ^)^. The decision to take medication or not is an empirical-rational strategy patients use to express their attempts to deal with the disease. It is important to acknowledge and not to underestimate the patients' decision-making abilities, identifying their beliefs and helping them adopt appropriate behaviors.

The Theory of Planned Behavior (TPB)^(^ 5 ^)^, one of the main models used to study health-related behaviors, assumes that beliefs impact predictive factors of intention (motivation) - immediate mediator of behavior. Hence, identifying beliefs regarding medication adherence is key to understanding patients' self-care actions because beliefs represent the opinions of patients, based on their knowledge or experiences, in regard to health orientation, influencing acceptance or rejection of the prescribed therapy^(^ 6 ^)^.

Even though TPB is widely used in studies seeking understanding various health behaviors, its application in studies addressing behaviors related to the prevention and/or control of DM is incipient. Among DM patients, there are studies addressing regular exercise^(^ 7 ^)^, insulin administration^(^ 8 ^)^, intake of low saturated-fat foods^(^ 9 ^)^, and adherence to oral antidiabetic agents^(^ 10 ^)^, however, no studies have been conducted with a Brazilian population with DM.

This study's aim was to identify and analyze salient beliefs - behavioral, normative, control and self-efficacy, related to adherence to oral antidiabetic agents through the application of TPB. Identifying these beliefs using TPB is an essential step to support the development of a measurement instrument to identify factors that determine adherence to oral antidiabetic agents. Understanding these determinants can aid the development of effective interventions to promote adherence to these medications.

## Method

Cross-sectional, exploratory study with a qualitative and quantitative approach guided by the TPB theoretical framework.

### Theoretical framework

This motivational model was derived from cognitive-social theories in which behavior is determined by *intention* (motivation) to act (effectively perform a given behavior) and by one's perception of control over behavior. Intention, the immediate antecedent of behavior, is determined by three factors: *Attitude* - an individual's assessment in regard to the probable or expected outcomes of a behavior; *Subjective Norm - *perceived social pressure, that is, the individual's perception concerning the opinion of social references in regard to a given behavior; and *Perceived Behavioral Control* - the individual's perception in regard to his/her control over behavior^(^ 5 ^,^ 11 ^)^. Each of the three determinants of Intention is formed by their respective beliefs. *Behavioral beliefs* refer to the assessment of results of behavior and produce a favorable or unfavorable attitude toward it. *Normative beliefs* refer to the perception of social references concerning a given behavior and result in a subjective norm. *Control beliefs *are created with the presence of perceived factors that may either facilitate or hinder a given behavior and influence perceived behavioral control^(^ 5 ^,^ 11 ^)^. The model recommends that, when identifying beliefs, salient beliefs, i.e., those that come first to mind when an interviewee is asked open questions about behavior, should be taken into account^(^ 12 ^)^.

Even though the literature indicates the strong predictive power of TPB in determining behavioral intention in regard to a complex range of behaviors, researchers^(^ 13 ^)^ have defended the inclusion of other variables as being able to improve the explanation of variability of motivation and its predictive power for behavior. The selection of these variables depends on their relevance to understanding a desired behavior and the characteristics of the studied behavior. In regard to medication adherence, the variable *Self-efficacy *was considered relevant and refers to an individual's confidence in his/her ability to perform certain behaviors that influence the events affecting his/her life^(^ 14 ^)^. 

### Study setting

This study was conducted in a general adult outpatient clinic of a large university hospital in the interior of the state of São Paulo.

### Subjects

Individuals 18 years old or older, with diagnosis of type 2 DM, under chronic use of oral antidiabetic medications for at least six months, monitored by the aforementioned clinic, participated in the study. Individuals whose administration of medication was managed by a caregiver, who used insulin, had been hospitalized or undergone surgery in the last 30 days, or showed impaired understanding or communication abilities, were excluded. A convenience sample was used. All the patients who met the inclusion criteria were consecutively added into the study until data saturation was achieved, i.e., when no new information was obtained and reports became redundant^(^ 15 ^)^.

### Data collection

Data were obtained from March to April 2012 through individual semi-structured interviews, under a free-response format intended to identify salient beliefs^(^ 11 ^)^. The interviews were conducted and recorded in a private environment and later transcribed verbatim.

### Instruments

- Socio-demographic and clinical characterization: composed of socio-demographic data (age, sex, schooling, work status, and family income) and clinical data (length of diagnosis, number of medications, and classes of antidiabetic agents being used);

- Self-reported measurement of medication adherence, according to two criteria^(^ 2 ^)^ - 1) *Proportion of adherence: *assessed through four frameworks that describe: 1. Name, dose and posology of the prescribed medication; 2. How each medication was taken in the 24 hours prior to the interview; 3. How medication was taken in the prior week; and 4. How medication was taken in the prior month. Tables 2 and 3, concerning medication intake for the day and week before, are intended to obtain more accurate responses through minimizing memory bias. Adherence to oral antidiabetic medications was calculated based on the doses omitted in the last month as reported by the patient according to the following formula [(prescribed doses - missed doses) x 100 / prescribed doses]^(^ 16 ^)^. For those taking more than one oral antidiabetic agent, the final proportion was computed by the mean of adherence percentages for each medication. The proportion of adherence was assessed as a continuous variable (mean of medication taken) and a categorical variable, classified into the appropriate number of doses (≥80% of the prescribed) and an insufficient number of doses (<80%); 2) *Global assessment of adherence* considers, in addition to the proportion of doses taken, the way medications were taken, the frequency, and care required for their administration, while considering association with temporal markers: fasting, breakfast, lunch, dinner and bedtime. The patients were classified into the groups: I - Number of doses and care appropriate to prescription; II - Appropriate number of doses and inadequate care; III - Insufficient number of doses and appropriate care; and IV - Inappropriate number of doses and care. The patients classified into Group I were considered to be adherent to medication treatment and those classified into Groups II, III and IV were considered non-adherent to their medication regimens.

**Table 1:** Sociodemographic and clinical characterization of patients with Diabetes Mellitus in outpatient follow-up by a university hospital (N=17). Campinas, SP, Brazil, 2012

Variable			n	%	Average (SD)	Median	Observed variation
Sociodemographic							
	Age (years)				59.8 (13.9)	57	40-84
	Schooling (years)				3.9 (3.3)	4	0-11
	Monthly family income (MW)*				2.5 (2.0)	1.9	1-8
	Sex – Female		11	64.7			
	Work situation						
		Active	1	5.9			
		Inactive/homemaker	16	94.1			
Clinical							
	Length of DM diagnosis (months)				68.6 (62.1)	48	8-240
	Number of classes of medication in use				6.8 (2.8)	7	3-13
	Oral antidiabetic agents in use						
		Metformin	8	47.1			
		Sulfonylurea	2	11.8			
		Metformin + Sulfonylurea	7	41.2			
	Adherence proportion (%)				83.7 (25.7)	95	0-100
		Use ≥80% of the prescribed doses	12	70.6			
	Global assessment of adherence						
		Adherent	9	53.0			
		Nonadherent	8	47.0			

* Minimum Wage (MW) current in April 2012 = R$ 622.00 (equivalent to USD 339.89)

**Table 2:** Behavioral beliefs concerning the behavior of adhere to oral antidiabetic agents. Campinas, SP, Brazil, 2012

Behavioral Beliefs		%
To take medication for diabetes treatment exactly as prescribed by my physician...		
	It causes side effects	82.4
	It keeps the glycemia and diabetes under control	58.8
	It prevents diabetes complications	52.9
	It prevents hospitalization and death	41.2
	It avoids or delays the need for insulin	29.4
	It causes hypoglycemic symptoms	29.4
	It eases hyperglycemic symptoms	23.5

**Table 3:** Normative beliefs concerning the behavior of adhere to oral antidiabetic agents. Campinas, SP, Brazil, 2012

Normative Beliefs		%
When taking medication for diabetes treatment exactly as prescribed, I would be approved by…		
	My children	70.6
	My physician	58.8
	My spouse	52.9
	The nursing staff	17.6
When taking medication for diabetes treatment exactly as prescribed, I would *not* be approved by…		
	Acquaintances who are diabetic and do not adhere to treatment	58.8

- Elicitation of beliefs concerning adherence to oral antidiabetic agents: developed according to the TPB^(^ 11 ^)^ assumptions and submitted to content validity by five judges who are experts in behavioral studies, in the application of the TPB, and in the construction and validation of measurement instruments^(^ 17 ^)^. The instrument is composed of 17 open and semi-open questions distributed into general beliefs concerning behavior (one), salient behavioral beliefs (three), normative (five), control (six) and self-efficacy beliefs (two). Behavior was defined as: "to take medication for diabetes treatment exactly as prescribed by my physician over the next two months", which includes target, action, context and time, in accordance with the TPB^(^ 11 ^)^ premises.

### Data analysis

The answers were submitted to content analysis based on the recommendations of the TBP theoretical model^(^ 11 ^,^ 18 ^)^. The following stages were followed: *i*) exploration of material in order to categorize it using categories pre-established by TPB, i.e., behavioral, normative, control and self-efficacy beliefs concerning adherence to oral antidiabetic medication; *ii*) the answers were grouped into each category according to themes or subcategories that were extracted from the participants' reports. The subcategories that emerged from the reports were submitted to inter-observer analysis to ensure reliability of assessment (two researchers who were experts in the application of TPB), with an agreement level of 95% between the judges, and *iii*) the frequency of subcategories were calculated and modal/more frequent beliefs were highlighted. To identify which beliefs would be included, we used one of the criteria proposed by TPB, in which beliefs that exceed a given frequency are considered modal. All the beliefs mentioned in this study by at least 10% of the sample were included. 

### Ethical aspects

The Project was approved by the Institutional Review Board at a university in the interior of São Paulo, Brazil (Process No. 6.608/2012).

## Results

The sample was composed of 17 individuals, most of whom were women (64.7%), aged 59.8 years old on average, with 3.9 years of schooling on average, and lived with other people (88.2%). Family income was 2.5 times the minimum wage on average (Table 1).

In regard to the prescription of oral antidiabetic agents, we verified the prescription of Metformin only (47.1%) or in association with Sulfonylurea (41.2%). The patients reported they took 83.7% of the prescribed doses in the prior months to the interview and most (52.9%) were considered adherent in the global adherence assessment.

Considering behavioral beliefs concerning adherence to oral antidiabetic agents, seven analytical subcategories emerged, representing the advantages and disadvantages of behavior of adhere to oral antidiabetic medication (Table 2). Disadvantages were mentioned in most of the reports, especially the medications' side effects (82.4%), followed by the hypoglycemic symptoms attributed to the medications (29.4%), as shown in the following reports: *It's not normal to have loose bowels so often. You may get dehydrated *(Subject 16).* Sometimes I get all sweaty, my tongue tingles. So I have to eat something salty or sweet. This always happens to me. It gets out of control. So, I guess it's because of the chronic use of medication *(Subject 16).

Advantages included having the disease and glycemia under control, which were mentioned in 58.8% of the reports, and preventing complications, highlighted by 52.9% of the participants, as the following reports show: *So, the medication controls sugar levels more than diet does. I'm not taking it these days and my sugar's increasing* (Subject 2)*; Now if I don't take it, later on I may have complications. I'm afraid of developing complications, yes, because there was one case in my family and I had to take care of this person. And she didn't take the medications *(Subject 16). The prevention of negative outcomes such as death and hospitalization, or obtaining positive outcomes such as delaying treatment with insulin and hyperglycemic symptoms relief were less frequently mentioned. The following excerpts respectively reveal these beliefs:* When my wife forgets, my daughters remind me. So I'm always reminded. They don't want me hospitalized because of lack of medication *(Subject 7)*; The physician said that if I responded well to this medication, I would not need insulin. So, I'd rather take the medication than insulin *(Subject 13)*; It helps yes, it eases my diabetic symptoms. The burning sensation in my feet. I feel better. It doesn't cure, but improves it *(Subject 10).

In regard to normative beliefs, the participants identified four groups of positive social references - their children, physicians, spouses, and the nursing staff, as shown in the reports: *One of my children always helps me. If they don't remind me, there's no way I will remember. I always forget. My memory has failed for some time now. It's more difficult *(Subject 8)*; I follow the physicians' recommendations, I trust them very much *(Subject 7)*; He *(points to the husband)*helps me, he fetches it for me. Because the health unit is kind of far away and I have this pain in my legs, so he goes and fetches it for me. 'Gimme the prescription' he says *(Subject 2)*. There're the nurses from the diabetics meetings. They say: This diabetes thing kills, you know? You may have to have your leg cut out, you cannot get hurt *(Subject 2). The presence of close family members (spouse, children) were considered favorable to adherence because they support the acquisition of medication, organize medications in appropriate containers, help the patient remember the times to take medications, and explain the therapeutic scheme to the patient.

The reports show that diabetic people who do not adhere to their medication treatment are referred to as negative social references: *There is a cousin of my wife who says: "my grandmother had diabetes since her 30s, never took medication and died at the age of 90. Forget about it". She says things like that and thinks I'm wrong because I'm forbidden of eating so many things. She thinks that I'm suffering for nothing, that, no, I don't need to take it *(Subject 7). These referents are those who experience complications due to DM, who do not adhere to their treatment, who report family problems, who do not care about their health, live by themselves, are either too old or too young, and most of all, do not believe in the treatment (Table 3). 

In regard to control beliefs, factors that either impeded/hindered or favored/facilitated behavior were reported and represent one and five categories, respectively. Among the five factors that facilitate are: the distribution of free-of-cost medication; having a routine and control of daily activities; taking medication associated with temporal markers; taking their medications with them whenever leaving home; and differentiating pills and tablets by color, form and size. The following reports reveal control beliefs:* Getting the medications at the health unit helps...Some days you don't have a coin to buy bread; I finish lunch and take the medication right after it, with a full stomach* (Subject 17);* Whenever I leave home, I take it with me. It's a habit already. It's like my wallet; I take it with me wherever I go out *(Subject 7);* I don't know the name, but I recognize it by the color and size. Some are all the same *(Subject 12)*; I have a schedule for everything, for breakfast, lunch, afternoon snack, dinner, everything *(Subject 4).

Taking medications more than once a day hinders adherence to antidiabetic treatment:* I have to take it in the morning and at night and this is what troubles me. It would be better if I had to take just once a day *(Subject 10) (Table 4).

**Table 4:** Control beliefs related to the behavior of adhere to oral antidiabetic agents. Campinas, SP, Brazil, 2012

Control Beliefs		%
These make it *easier* to take medication for diabetes treatment exactly as prescribed…		
	Access medication free-of-cost	70.6
	Associate time of medication with temporal markers	29.4
	Bring the pills whenever leaving home	29.4
	Being able to differentiate pills by color, form and size	17.7
	Having a routine and control over daily activities	11.8
This makes it *harder* to take medication for diabetes treatment exactly as prescribed…		
	Having to take medication more than once a day	23.5

Self-efficacy beliefs refer to the individual's perception concerning his/her own ability to perform a given behavior. Analysis of this excerpt - *The only thing I was talking about is that there were medications I was taking that I didn't even know what they were for [...] But, once I understand what they were for, things get easier *(Subject 13) - reveals that the factor that enabled this individual to perform the behavior was understanding the prescription. A belief that explains a possible inability of an individual to adhere to oral antidiabetic medication is forgetfulness, as the following report shows:* [...] when I remember, you know? I work and take the medication with me. The problem is that I forget, I become involved with my work *(Subject 14) (Table 5).

**Table 5:** Self-efficacy beliefs concerning the behavior of adhere to oral antidiabetic agents. Campinas, SP, Brazil, 2012

Self-efficacy beliefs		%
I feel capable of taking medication for diabetes treatment exactly as prescribed due to...		
	Understanding the prescription and how the medication should be used	35.3
Sometimes I don’t feel capable of taking medications for diabetes treatment exactly as prescribed due to…		
	Forgetfulness	17.6

## Discussion

This study's aim was to elicit and analyze salient beliefs concerning adherence to oral antidiabetic agents through the application of TPB. This is a pioneering study in the Brazilian context, the findings of which can support the development of an instrument to measure the determinants of adherence to oral antidiabetic medication. According to TPB, each of the cognitive determinants of behavior consists of a set of beliefs and individuals' assessment of such beliefs. Therefore, identifying the reasons behind the use of oral antidiabetic medication is key to understanding the determinants of medication adherence, as well as to supporting the design of effective interventions to promote this behavior among DM patients^(^ 19 ^)^. 

Health professionals face the challenge of discovering the cause of hyperglycemia when dealing with patients whose DM is poorly managed; that is, the professional has to determine whether the poor management is related to treatment nonadherence or occurs despite the correct use of medications. Since patients may be more willing to report their negative beliefs concerning medication treatment than admit to low adherence, asking for their beliefs may enable the identification of patients with a greater likelihood of nonadherence to treatment. 

In regard to behavioral beliefs, we highlight the impact of perceptions of reactions attributed to oral antidiabetic agents, which may lead to nonadherence. This result was also found in previous studies^(^ 19 ^-^ 20 ^)^. Hypoglycemic episodes are also disadvantageous to adherence, responsible for irritation, sickness, and weakness, which contribute to the poor glycemic control of DM^(^ 21 ^)^. Fear of these events increases stress associated with DM, with an important impact on the management of the disease and metabolic control^(^ 22 ^)^. The advantages of adherence are related to controlling the disease, to minimizing complications, and preventing negative outcomes, which demonstrates the perception of DM as a progressive, potentially fatal, disease that culminates in treatment with insulin and impacts quality of life. A similar study^(^ 10 ^)^ reports verbal accounts of a feeling of wellbeing that accrues from the regular use of oral antidiabetic medication, the potential reduction of complications, and maintaining control over glycemia and DM. 

In regard to normative beliefs, positive social referents included the patients' children, physicians, spouses, and the nursing staff. Diabetic patients who do not adhere to the medication scheme were considered negative social referents. Health professionals should consider the influence of social references in the motivation of diabetic patients to adhere to treatment and propose interventions that promote positive referents. A previous study^(^ 23 ^)^ using TPB in the identification of beliefs related to adherence to anti-retroviral therapy among seropositive Latin immigrants reports that family, spouse/partner, physicians, and seropositive friends, in addition to the HIV/AIDS counselors and members of HIV support groups, were considered positive social references.

In regard to control beliefs, the factor that hindered or impeded medication adherence was the need to take medications more than once a day. It shows that the complexity of the therapeutic scheme is a factor that does not favor the correct use of medications. The factors that favored or facilitated adherence behavior were having access to free-of-cost medication, following a routine and having control over daily activities, taking medication associated with temporal markers, carrying medication when leaving home, and differentiating pills and tablets by color, form and size. Such items reveal that medication adherence is supported by ease of access to medication and by incorporation of medication into a daily routine, also a finding observed in previous studies^(^ 10 ^,^ 24 ^)^.

Finally, positive beliefs in regard to self-efficacy in treatment adherence were: understanding the prescription and how the medication is used. The negative belief in this category was forgetfulness. These data corroborate findings in the literature^(^ 20 ^-^ 25 ^)^. A similar study^(^ 19 ^)^ identified most of the beliefs concerning DM medication adherence as being related to low adherence, highlighting the perception that there is no need to take medication when glycemia is under normal levels, concern over side effects, or due to complexity of the therapeutic scheme and dependence in following it. 

## Conclusion

This study's results show that adherence to oral antidiabetic agents is a complex behavior permeated by behavioral, normative, and control beliefs, which should be taken into account when assessing determinants of behavior. Behavioral beliefs concerning the perception of adverse reactions, hypoglycemia, the possibility of controlling glycemia and the disease, and prevention of negative outcomes emerged. Social references included the patients' children, physicians, spouses, the nursing staff and other diabetic patients. Having access to free-of-cost medication, following a routine and having control of their daily life, taking medications associated with temporal markers, carrying medication whenever leaving home, and being able to differentiate pills by color, form and size facilitate adherence. On the other hand, having to take the medication more than once a day hinders adherence. Understanding the prescription undergirded the ability to perform the behavior while forgetfulness explained a possible inability to adhere to treatment. Investigation concerning the influence of beliefs in regard to the intention to adhere to oral antidiabetic agents can support interventions focused on the promotion of medication adherence among DM patients.
